# The Ethics and Politics of Academic Knowledge Production: Thoughts on the Future of Business Ethics

**DOI:** 10.1007/s10551-022-05243-6

**Published:** 2022-09-26

**Authors:** Gibson Burrell, Michael R. Hyman, Christopher Michaelson, Julie A. Nelson, Scott Taylor, Andrew West

**Affiliations:** 1 Alliance Manchester Business School, University of Manchester, Manchester, UK; 2 Institute for Marketing Futurology and Philosophy, Las Cruces, USA; 3 Opus College of Business, University of St. Thomas, Saint Paul, USA; 4 College of Liberal Arts, University of Massachusetts Boston, Boston, USA; 5 Birmingham Business School, University of Birmingham, Birmingham, UK; 6 School of Accountancy, Queensland University of Technology, Brisbane, Australia

**Keywords:** Institutionalisation, Practices, Stories, Peer review, Marketing theory, Feminist economics, Paraethics, Future of business ethics

## Abstract

To commemorate 40 years since the founding of the Journal of Business Ethics, the editors in chief of the journal have invited the editors to provide commentaries on the future of business ethics. This essay comprises a selection of commentaries aimed at creating dialogue around the theme *The Ethics and Politics of Academic Knowledge Production.* Questions of who produces knowledge about what, and how that knowledge is produced, are inherent to editing and publishing academic journals. At the Journal of Business Ethics, we understand the ethical responsibility of academic knowledge production as going far beyond conventions around the integrity of the research content and research processes. We are deeply aware that access to resources, knowledge of the rules of the game, and being able to set those rules, are systematically and unequally distributed. One could ask the question “for whom is knowledge now ethical’”? (See the Burrell commentary.) We have a responsibility to address these inequalities and open up our journal to lesser heard voices, ideas, and ways of being. Our six commentators pursue this through various aspects of the ethics and politics of academic knowledge production. Working with MacIntyre’s scheme of practices and institutions, Andrew West provides commentary on the internal good of business ethics learning and education. Inviting us to step out of the cave, Christopher Michaelson urges a clear-eyed, unblinking focus on the purposes and audiences of business ethics scholarship. As developmental editor, Scott Taylor uncovers some of the politics of peer review with the aim of nurturing of unconventional research. Mike Hyman presents his idiosyncratic view of marketing ethics. In the penultimate commentary, Julie Nelson attributes difficulties in the academic positioning of the Business Ethics field to the hegemony of a masculine-centric model of the firm. And finally, Gibson Burrell provides a powerful provocation to go undercover as researcher*-*investigators in a parallel ethics of the research process.

## Business Ethics Institutionalised: Prospects for Learning and Education

**Andrew West**

### Introduction

There is little doubt that, over recent decades, ‘business ethics’ has increasingly become mainstream, both as an academic discipline and as a focus area for business. Scholars in the field have a choice of conferences to attend and journals in which to publish, universities teach courses in business ethics, embed business ethics throughout programmes and offer professorships and chairs in business ethics and related fields. Accrediting agencies, such as the Association to Advance Collegiate Schools of Business (AACSB), EFMD Global (EQUIS), and AMBA, alongside various industry-specific professional bodies, support, and/or require ethics content in business programmes. Universities commit to initiatives such as the Principles of Responsible Management Education and the UN Global Compact.

Many businesses have ethics officers and formal ethics training programmes for their employees; corporate and professional codes of ethics are common; corporate social responsibility initiatives and reporting are prominent. Formal statements, guidelines, and frameworks, ranging from the Caux Round Table Principles, Global Reporting Initiative, Principles for Responsible Investment and various corporate governance reforms around the world have ensured that ethical considerations in business are visible and permanent.

It might appear, therefore, that the task of establishing business ethics as an academic discipline and of embedding business ethics within business has been completed. Serious ethical problems relating to business remain, of course (from ongoing fraud and corruption to environmental sustainability, inequality, modern slavery, and many others), and there is a continuing need for research that adopts different perspectives to further our understanding of these problems. However, in this commentary, I suggest an alternative viewpoint. Drawing on Alasdair MacIntyre’s work, I draw attention to a conflict inherent in the process of institutionalisation, while also offering insight into possible remedies. In this vein, I provide a rudimentary application of MacIntyre’s scheme of practices, institutions, and internal and external goods to highlight issues relevant to the future of business ethics scholarship and to the field of business learning and education in particular.

### MacIntyre’s Scheme of Practices and Institutions

MacIntyre, in his most well-known work, *After Virtue*, proposed a sociology in which the good is located primarily in practices. He described these practices as:

any coherent and complex form of socially established cooperative human activity through which goods internal to that form of activity are realized in the course of trying to achieve those standards of excellence which are appropriate to, and partially definitive of, that form of activity, with the result that human powers to achieve excellence, and human conceptions of the ends and goods involved, are systematically extended. (2007, p. 187)

He provides examples as varied as painting, architecture, medicine, chess, farming, and football. These practices encompass internal goods (such as excellent painting or responding creatively to problems) that can only be obtained within the practice. They are contrasted with external goods of wealth, power, and status: goods that are the objects of competition and are of limited supply. While it is within such practices that practitioners are morally educated, and where they develop traits such as courage, justice, and truthfulness, the practices are nevertheless vulnerable. Practices are necessarily housed in institutions, and institutions necessarily deal in the external goods of wealth, power, and status, such that the “ideals and creativity of the practice are always vulnerable to the acquisitiveness of the institution, in which the cooperative care for common goods of the practice is always vulnerable to the competitiveness of the institution” (p. 194).

Despite this relationship between practices and institutions, external goods remain goods, and MacIntyre does not suggest that they are to be avoided. Rather, it is when external goods are prioritised over internal goods that difficulties are likely to arise. This provides one means of characterising some (if not most) corporate ethical failures, and MacIntyrean scholars have indeed found this a useful framework for analysing ethics in business (Beabout, 2020; Beadle, 2017).

This lens can also be used to consider business ethics as an academic discipline. We could view ‘business ethics scholarship’ as a MacIntyrean practice (while it is beyond the scope of this commentary to provide a more in-depth analysis, arguments could be made both for and against) and suggest that its internal good consists of quality research that extends our understanding of ethics in business, with concomitant impacts on businesses themselves. Such a good is both individual and communal. As practitioners engaging in this practice, we develop traits such as perseverance, truthfulness, discipline, and, when coping with rejection letters or encountering those with more experience in the field, humility. The production of quality research informs, challenges, critiques, and extends the community of business ethics scholars, while contributing towards an understanding of good business activity that has practical implications. This community of scholars works within (and extends) shared standards of excellence associated with quality research, includes novice apprentices as well as experts in the field, and incorporates training and learning by which novices are introduced to these standards of excellence.

My purpose in casting business ethics scholarship as a MacIntyrean practice is to highlight how, given the institutionalisation of business ethics over recent decades, the tension between practices and institutions is of particular relevance. We are led to question whether this practice of business ethics scholarship is being, or will be, corrupted. To what extent is pursuit of the internal good of quality research subservient to the pursuit of the external goods of wealth, status, and power?

Reputation and status are clearly of great importance in academia—for universities, schools, and departments, as well as individual researchers. To the extent that reputation and status constitute the aims and ends of scholarly activity, the risk of the internal good (quality research) being corrupted increases: if a researcher or university is motivated primarily by external goods and could achieve their desired reputation and status with compromised research quality, they would have no reason not to do so. At the same time, academics and university administrators are under constant and apparently increasing pressure to meet reputational targets: publishing a prescribed number of articles in top-tier journals over a particular time frame, attracting a given quantum of research funding from industry sources, reaching a certain band in university ranking exercises, etc.

The institutionalisation of business ethics scholarship means that the questions we now pose for the future of business ethics scholarship are not limited to issues and perspectives that have thus far been under-explored, or about those that are of particular urgency. In MacIntyre’s scheme, while wealth, status, and power remain goods (and increased reputation and status may be associated with quality business ethics scholarship), practices need to be safeguarded by practitioners pursuing internal goods, and we accordingly need to consider how we retain the integrity of business ethics scholarship and resist its capturing or commodification in the future. How might we retain a focus on the internal good of quality research?

A partial answer to this question lies in the motivation of scholars, administrators, and publishers. This, in turn, is related to an individual’s life projects and their own personal narrative (particularly with regard to academic careerism). We might be so bold as to ask what motivations scholars ought to have, and whether some desires related to academic publishing may be misdirected. Indeed, for MacIntyre, the corruption of practices is associated with the exercise of vices, and their protection with the exercise of virtues, typically including courage, justice, and truthfulness. However, practices in good order are also characterised by ongoing reflection on their internal goods and standards of excellence. So, another partial answer lies in (re)considering the internal goods and standards of excellence involved in the practice of business ethics scholarship, what we understand by quality research in business ethics, and its ends and goals (see Michaelson’s and Burrell’s commentaries in this essay). This provides us with an opportunity to re-focus the attention of the community of practitioners on the status of the practice of business ethics scholarship and provides a bulwark to the corrupting influence of its institutionalisation.

The remainder of this commentary is the beginning of such an exercise, but with a focus on the subdiscipline of business ethics learning and education scholarship. I reflect firstly on the internal good associated with scholarship in the field of business ethics learning and education and subsequently on the standards of excellence associated with the Business Ethics Learning and Education section of the Journal of Business Ethics.

### The Internal Good of Business Ethics Learning and Education Scholarship

When reflecting on the internal good associated with business ethics learning and education scholarship, we can begin with the terms themselves. A useful starting point is Illeris’ (2007, p. 3) broad definition of learning as “any process that in living organisms leads to permanent capacity change and which is not solely due to biological maturation or ageing”. Education, as the processes that facilitate such learning, points to its sociological, relational, cultural, and political context. Turning to these ‘first principles’ allows us to place our existing scholarship. That is, research in business ethics learning and education very often focuses on learning by individuals in higher education institutions. Furthermore, a great deal of this research has concentrated on whether particular interventions within university courses have had an impact on how students form judgements on particular ethical issues (Medeiros et al., 2017; Waples et al., 2009). While these investigations are (and will continue to be) relevant, to the extent that we limit our focus to this type of learning and education, we are at risk of unnecessarily limiting our understanding of our own practice, and thereby restricting its impact.

Since business ethics as a field has an explicit focus on improving society (whether in our understanding of ethics in business, or within businesses themselves), how we learn about ethics in business is a question of paramount importance. Anybody who has any involvement in business has views on whether particular business activities or structures are good or bad, right or wrong. These views may not always be articulated, they may not always be reflected upon critically, but they are nevertheless learned, and many are learned outside of the university classroom. When considering the internal good, and what constitutes quality research that extends our understanding of business ethics learning and education, we ought therefore not limit ourselves, and our investigations in this field must necessarily be cast wider. I suggest five inter-related ways in which this may develop, each of which is intended to extend our conception of the internal good of quality research in business ethics learning and education.

Firstly, we can extend our focus beyond individual decision-making. This is not to downplay the importance of understanding how individuals learn to reason and come to ethical judgements, but to emphasise the importance of other aspects of learning that are all too easily overlooked. This can include focusing on conative and affective dimensions, alongside the cognitive. Building on work that has explored the links between emotions and ethics, for example, there are questions around how this plays out in the learning process. Similarly, if we know that coming to an ethical decision does not always result in ethical action, how do we actually learn to behave ethically? In learning about ethics in business, what role is played by motivations, attitudes, desires, past experiences, dispositions, and character traits? How does learning business ethics relate to one’s life story and involve biography? What role does tacit knowledge play?

Secondly, we can consider how learning can be transformative. While there are multiple ways in which this can be interpreted, if education in business ethics is to do more than reaffirm one’s existing outlook, we expect it to challenge. Unlike learning accounting techniques or principles of marketing, ethics education is intensely personal. Mezirow’s (2006) concepts of a “disorienting dilemma”, critical self-reflection in relation to one’s assumptions and dialectical discourse all have a particular applicability to ethics education, which is necessarily engaged with personal values, and the reasons, experiences, and hopes that they draw upon. Transformation and challenge can also refer to the context of business and we can ask whether business ethics education does (or ought to) adopt a critical stance—in terms of exposing power structures, injustices, and inequalities in different types of businesses and economic systems. To what extent does business ethics education have (or ought to have) a focus on action for social change?

Thirdly, ethics education can, and ought to be, inclusive, particularly in terms of diversity of thought and an openness to alternative perspectives. Much discussion of business ethics learning and education assumes a secular Western heritage and Western institutions. To the extent that our enquiries are confined in this way, we limit our understanding and run the risk of developing a narrow-minded and impoverished conception of how business ethics can be learned (see Taylor’s commentary in this essay). How is business ethics learned (or taught) in non-Western or non-secular contexts? To what extent do learners’ different cultural backgrounds influence their learning of business ethics, and how? In a pluralist context, how do learners deal with conflicting viewpoints?

Fourthly, education is not always accredited, and learning is not restricted to formal settings in educational institutions. Business ethics can be learned in a multitude of environments, including the family, schools, community groups, religious organisations, workplaces, and professions (perhaps suggesting “communities of practice”). Business ethics can be learned later in life, through periods of crisis, and in childhood. Reflecting on the scope of business ethics learning and education scholarship involves a broader consideration of formal learning (structured within formal educational institutions or programmes), non-formal learning (structured learning outside of formal educational institutions or programmes), informal learning (unstructured learning), and self-directed learning. How these areas relate to each other and how business ethics learning is transferred from one context to another also require consideration.

Lastly, we need not limit our enquiries into how business ethics is learned. Understanding the barriers to learning business ethics and how ethics may be ‘mis-learned’ also contributes to developing our understanding of the field. This may occur in various ways, including individually (such as due to personal ambivalence), through social influence, and within particular organisational or institutional structures. We can also extend our enquiry to include understanding how *unethical* behaviours, attitudes, values, and beliefs are learned, and the conditions that facilitate (or hinder) such learning.

Each of these five ways of developing scholarship in business ethics learning and education reconsiders and extends our conception of the internal good of quality research in this area. The list is not intended to be exhaustive, and ongoing reflection may (and should) suggest further avenues to explore. However, this process of reflection points to exciting opportunities by which the practice may productively develop (ways that are particularly well suited to business ethics—the application of each of these areas is far more limited in other more technically or vocationally oriented business disciplines), and how it may contribute further to the common good of better business activities and business structures. If MacIntyre is right, ongoing reflection and debate on the nature of this practice, and appreciation of how scholars can make valuable contributions, help us to maintain a practice in good order and put us in a better position to resist the corrupting influence of academic institutionalisation.

### Learning and Education at the Journal of Business Ethics—Standards of Excellence

Turning to the standards of excellence related to scholarship in business ethics learning and education, I observe firstly that academic work (and therefore excellence) in this area can take a variety of forms, including the development of university textbooks, curricula and learning materials, self-reflections on teaching practice, preparing guides for industry, as well as academic research outputs. The Business Ethics Learning and Education section within the Journal of Business Ethics is limited to the last of these, and its purpose is to further our academic understanding of business ethics education and how business ethics is learned in any and all contexts. As indicated in the reflection above, the scope of research can be broadened in multiple ways; so, while research on rational approaches to university students’ decision-making is not in any way discouraged, alternative approaches and wider perspectives are encouraged. Regardless of the topic, perspective or approach adopted, as practitioners in the practice of business ethics learning and education scholarship, we aspire to (and share) certain standards of excellence, and I draw attention to two of these in particular.

First is the oft-quoted need for research that makes a theoretical contribution. As the aim of this section is to further our understanding, papers are expected to engage seriously with, and extend the theories, concepts and/or philosophical perspectives through which this understanding is expressed. To contribute to our aim, all papers should enhance our understanding of business ethics learning and education in some way, such that we can easily point to what it is that we now know, and that we did not know before. It follows that papers that aim primarily at proposing educational resources, sample curricula or that provide speculations on teaching practice are unlikely to achieve excellence in this context.

The second involves the quality of argumentation (see Hyman’s commentary in this essay). All papers that make a theoretical contribution further our understanding by means of an argument. For empirical papers, this may be bound up with the typical structure of such a paper and the methods adopted, and rigour is required for the conclusions to be convincing. For conceptual papers, however, considerable attention needs to be paid to ensuring that the argument is clear. This requires articulation of the reasons that support the conclusion, and a critical argumentation that considers counterarguments and objections.

Despite the possible adverse consequences of an increasingly institutionalised business ethics (and business ethics scholarship) that are suggested by a MacIntyrean interpretation, there are nevertheless good reasons to remain positive. Although business ethics scholarship (as a MacIntyrean practice) may be subject to corruption, there are ways of resisting such corruption and maintaining a practice in good order. One way of contributing to this is through ongoing reflection on the practice’s internal goods and standards of excellence. By doing so in relation to business ethics learning and education scholarship, I have proposed five ways in which we may reconceive the internal good and emphasised two standards of excellence associated with academic scholarship. I hope that this in turn can stimulate more quality research in business ethics learning and education, a good both individual and communal.

## Business Ethics Out of the Cave

**Christopher Michaelson**

### Who is in the Cave?

Many readers of this journal have at least a passing familiarity with Plato’s famous cave allegory, in Book VII of the *Republic*. Arguably one of the most influential passages in the history of human thought, it describes a group of prisoners, chained to the ground facing forward, who can see only shadows of moving objects behind them, cast on to the wall in front of them by the light from a fire still farther behind. When one of them breaks free and discovers the real world outside the cave, he is ridiculed by the remaining prisoners who have come to believe that the shadows are reality.

When read in full, the story not only establishes the philosophical foundations of justice but also prefigures the psychological foundations of behavioural ethics. Arguably, it has underappreciated importance for business ethics, helping to explain, for example, why managers are sometimes insensitive to the reality of poor labour conditions of distant shop-floor workers. When maltreated human capital are just numbers on a balance sheet and their daily misery is concealed in a managerial blind spot, their concerns are only as real to management as if they were abstract shadows on the wall of a cave. The allegory also illuminates, for example, why people who work in carbon-intensive industries have an incentive to deny climate science. Just like the prisoners are loath to believe there is a world beyond theirs that disrupts the reality on which they depend, managers may be motivated to maintain an unsustainable revenue stream that has sustained them. In the allegory as Plato intended it, the philosopher is responsible for revealing the ignorance of the prisoners, much as we business ethics scholars might believe our job is to expose bad business behaviour and make it better.

However, great stories are usually receptive to multiple interpretations that are worthy of consideration. Accordingly, alongside that conventional application of the cave allegory, in which business actors are in the cave and scholars are in the sun-soaked reality, I would like also to try to turn our understanding of the allegory around. In this alternate reading, we scholars are the prisoners, locked in an unreal world. In this telling of the tale, business ethicists are not always the truth-bearers who have come to rescue the world from the shadowy unreality of business. Rather, the suntanned heroes in this romanticised version of the story are the entrepreneurial spirits and captains of industry who provide access to capital, build bridges, feed the hungry, cure diseases, and connect us technologically. Certainly they sometimes get greedy and make mistakes, but at least they do something in the real world. Meanwhile, imprisoned in our cave, also known as that echo chamber called the ivory tower, we academics cast stones at shadows upon the wall as though it makes a difference to the apparitions of commerce before us. Behind us, beyond the mouth of the cave, practitioners proceed with their real work, oblivious to our shadowy academic existence. The purpose of this commentary is to entertain alternate takes on the cave allegory, because reflecting upon its multiple meanings might help us business ethics scholars engage with the business world.

### How Business Ethics Matters

**Fig. 1:**
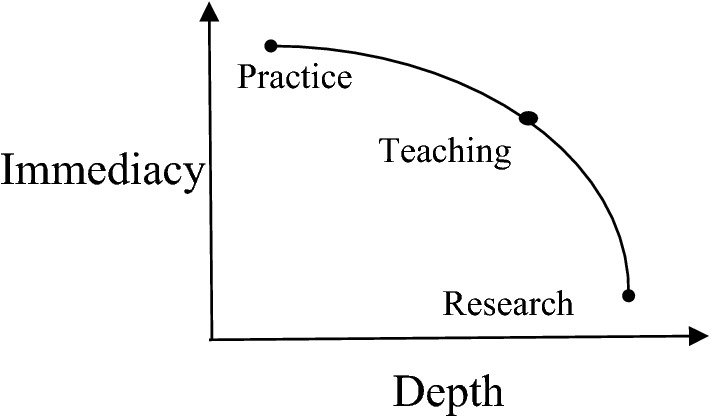
Impact of business ethics activities

Business is an ethically challenging and challenged social institution with significant power to make the world around it better or worse. As a business ethics scholar whose career began in the New York office of a Big Four professional services firm, I once sought to influence business from the within before seeking to do so from without. This has led me to wonder, in the parlance Iris Murdoch used to describe Plato’s cave allegory, which work—business or the scholarly examination thereof—has been closer to the fire and which has been closer to the sun. It seems to me now that this question is not unlike what I meant to explore in my first lecture as a professor who had returned to the business classroom after several years in the boardroom. I sketched a rougher version of this visually (see Fig. 1) to explain to my students why I had exchanged my old job, in the world in which many of them aspired to work, for my new job, on a campus from which they sought to escape.

Using what I had learned in management consulting about the explanatory value of two-dimensional matrices—a language which I would come to learn was also spoken by management academics—I asserted that the impact of our work upon the world could be measured in two dimensions: immediacy and depth. Immediacy is the rate at which our work influences the world. Depth is the amount and endurance of that influence.

In this representation of reality, practice has high immediacy and low depth. For example, I learned that senior executive attention to managing ethics and compliance programmes was typically temporary and sometimes superficial. Often provoked by crisis, these projects were designed quickly to mitigate damage so that the organisation could redirect its attention to its central priorities. Those priorities pertained to the company’s reason for being, the products or services that were its source of profitability and that are sometimes characterised as an organisation’s moral purpose.

At the other end of the matrix, research has low immediacy and, we hope, high depth. I can clearly remember only a few times in nearly 20 years when academic research and client work intersected. One was when Lynn Sharp Paine’s (1994) *Harvard Business Review* article distinguishing between “compliance strategy” and “integrity strategy” was institutionalised within the United States Federal Sentencing Guidelines for Organizations, ten years after her paper was published. Another was when I heard Klaus Schwab (2021) of the World Economic Forum speak around 2005 about stakeholders, a term which had been academically coined at least 25 years earlier. Even then, significant time had passed between academic publication and business practice.

My early days as a lecturer who kept a foot in the business world are still partially fired by the glowing embers of memory. My students admired me because they regarded me as though I were a visitor from the outside world who still maintained a sunny window office in the city with a spectacular view of the Empire State Building. Perhaps to justify to them—and to myself—why I accepted a significant pay cut to spend time persuading them that there was more to their future professional success than pay, I put teaching at the apex of a curve running between practice and research. What they learned in the classroom would influence their business practices after they graduated. But was my cavernous classroom a cave they were leaving, or were they about to be swallowed by the mouth of the business cave?

### How Business Ethics Scholars Communicate

As I have suggested, there are good reasons to believe that business managers are the prisoners of the cave allegory, so entranced with the fleeting riches before their eyes as to be insensitive to the eternal goods in the world behind them. However, my purpose here as an editor is to challenge business ethics scholars with the alternate possibility that we have been understanding the story backward, and that we scholars are the prisoners. Even if this alternate reading turns out to be wrong, one of the lessons of the cave allegory is to at least entertain the possibility that the worldview which we scholars have long taken for granted may be nothing more than a cheap imitation of the genuine article.

The logical leap is not difficult to make if we give it a try. The fundamental premise of the allegory is that the world behind the prisoners consists of things, whereas the world in front of them contains mere representations of things. Similarly, the world on which business ethics scholars reflect is one of things, such as people, manufactured products and their component parts, skyscraper offices and shop-floor workstations, and container ships transporting goods from place to place for production and consumption that are the basic functions of a supply and demand economy. The world of scholarship, by contrast, is a world of representations of things, such as empirical studies in which the things of business are represented by numbers and descriptors, conceptual theories about business things, and labouratory experiments that examine the perspectives of students who learn about business from theories and simulations. Scholars are people who ply our trade primarily with words—uttered in classroom teaching, conference presentations, and written articles—that are themselves mere representations of things. For millennia, scholars in the Western tradition have supposed that the things of business belong in the sensible world and the ideas of scholarship resemble the world of forms, but at least it is worth wondering whether it could be otherwise.

Our academic commitment to these shadows of reality is underscored by our quantitative measures of success, principally the much maligned but ubiquitous impact factor that not only is merely a shadow cast by our real papers but also serves to signal the influence of our work only within this world of scholarship, not on the world outside. Meanwhile, the primary qualitative barrier to our production that demarcates publishable from unpublishable research is arguably more concerned with methods than meaning. That is to say, it is much easier to publish ideas that incrementally build upon existing paradigms than it is to publish novel, paradigm-shifting ideas and methods. If you doubt that last assertion, consider whether Plato’s cave allegory, any chapter of Kuhn’s *The Structure of Scientific Revolutions*, or this editorial commentary that you are now reading for that matter, would make it past a desk editor and three peer reviewers to publication in most modern management journals.

Most of our words, which are crafted to achieve affirmation in this insular cave of scholarship, are as Greek to the managers whose behaviour we presume to enlighten as the original Greek of the cave allegory itself. In this way, the form of our scholarly output is only accessible to the objects of its inquiry in translation. Moreover, the detachment of our writing from the world outside is not only a problem of content but also of form. It begins with our problematisation—not to mention our use of long words like “problematisation”—which begins *in the literature*—meaning in the world of scholarship—rather than *in the world*—meaning in the world of business that we purport to write about.

### Practical Suggestions for Reaching Our Audiences

Ironically—because Plato was deeply suspicious of art—the cave allegory is a miniature masterpiece of literary artistry that illuminates the difference between wisdom and ignorance, and between the world and the shadows thereof. It also depicts the challenges that we face in recognising ignorance on the road to wisdom and in overcoming the literal and figurative distance between reality and representation. For business ethics as an applied field, these challenges are analogous to the gap between the way things work in the business world and our idealised management models—or, to put it in the simplest if not overly simplistic terms, the distance between practice and theory*.* Perhaps the cave allegory as it was intended to be read challenges scholars to descend back into the cave to share our wisdom with business managers. If that is so, then, in order to have an impact on practice, we will have somehow to entice them to follow us out of the cave into the sun. At least, this implies some kind of scholarly obligation to engage with practice, not merely to observe it from a safe distance but rather to empathise with the experience of the pressures and expectations that yield the kinds of behaviours that we scholars seek to refine.

However, that reading has seemed to me, since I wrote a doctoral dissertation many years ago called *Philosophy Out of the Cave*, arrogant and unlikely to win supercilious scholars many apostles among practitioners. Accordingly, as an editor of two sections of this journal (Arts, Humanities, and Business Ethics, and Book (and More) Reviews), my aim is to use stories and interpretations thereof to bring scholarship and practice closer together. These stories may include, for example, a creative reading of Plato’s cave allegory. More generally, these sections interrogate objects and techniques of aesthetics and art criticism to imagine “things such as might happen,” as Aristotle says poetry enables us to do. Here I would like to illuminate at least three ways in which engaging with fictional worlds of the arts and humanities can ironically help business ethics scholars engage with the real world of business.

One way is that *fictional narratives help us to understand nonfictional people better*. In their 2017 book, *Cents and Sensibility*, which takes its punny title from Jane Austen, Gary Saul Morson and Morton Schapiro (2017) argue that everything that modern behavioural economics has taught us about human irrationality has long been understood by great novelists. Novels demand that we immerse ourselves in the whole lives of their characters, cultivating the ability to “think ethically in the novelistic way” (p. 11). For example, I have proposed that there is no better text than Mary Shelley’s 1818 novel, *Frankenstein*, for accessing the mind of the modern social media entrepreneur who, enamoured of their ingenious creation, neglects to anticipate and manage its capacity for destruction. Alongside *Frankenstein*, I have called Mohsin Hamid’s (2007) novel, *The Reluctant Fundamentalist*, one of the most important novels of contemporary capitalism in its depiction of a young professional searching for his purpose. The novel’s protagonist, Changez, speaks in the second person to “you,” seating the reader across from the narrator at a dinner table in Lahore that brings you into a kind of Platonic dialogue with him, closer to any research subject than you are likely otherwise to get.

*Stories engage us in other minds, worlds, and interpretations by transporting us there*. For example, a literary critical reading of the cave allegory not only enables us to imagine a reversal of its central metaphor but also to bring the cave with us into the modern context in which we are reading it. Here we might twist the metaphor yet again into a management–labour story, in which we encounter the prisoners of contemporary capitalism with access only to shadows of the spoils enjoyed by management. Those prisoners include victims of three of the most significant tragedies of our young century: 9/11, the Great Recession, and the COVID-19 pandemic. Two recent novels, Laila Lalami’s (2019) *The Other Americans* and Ayad Akhtar’s (2020) *Homeland Elegies* suggest that the roots of political division in our world today can be traced in part to post-9/11 racial and religious discrimination. *Sweat*, a 2017 play by Lynn Nottage (2017), and *Behold the Dreamers*, a 2016 novel by Imbolo Mbue, put us in contact, respectively, with steelworkers in a poor industrial town who lose their jobs in the Great Recession, and an immigrant chauffeur whose family is economically dependent on a laid-off Lehman Brothers executive. The HBO television series *Station Eleven*, based on Emily St. John Mandel’s (2014) novel of that title, examines many missteps—many of them motivated by money—that allow a global pandemic to change human civilisation, while Ling Ma’s (2018) novel, *Severance*, follows the lives of surviving consumers touring the hollowed-out relics of capitalist offices and shopping malls. Fictional narratives that induce us to imagine possible worlds enable us to reimagine reality.

Finally, by making us better consumers of great stories, *studying stories can make us better storytellers*. This includes caring about the quality and engagement of our writing that makes it accessible and memorable not only to our fellow scholar-prisoners but also to those practitioner-philosophers in the world outside whom we hope to influence. It also includes unchaining ourselves from the conventional journal article that has become the standard form of the size and shape of a recognised academic idea—to try something different, such as the critical examination of artworks as symbols of economic status or a review of recent books, films, or television series about business ethics. Some of our worthy ideas may require but a Tweet, others a commentary like this, and still others might demand a book or more. Some may be more felicitously expressed in a podcast rather than a paper. Better storytelling can be cultivated by being more attuned to the narrative arts and humanities that can constitute the closest thing to the pieces of life with which we might aspire to engage.

## Development Editing and the Ethics of the Peer-Review Process

**Scott Taylor**

### Introduction

Double-blind peer review, with neither submitting authors nor reviewers aware of each other’s identity, has become a marker of a journal’s quality, integrity, and fairness in our field. As a process, it has become a standard practice to the degree that we are suspicious of journals that do not operate within it. It is often ethically justified on the basis of equality of access—anyone can submit their work, and all work submitted will be treated in the same way—with the implication that the best research will be published whoever has submitted it. However, we know from both qualitative and quantitative evidence that peer review is politicised as a process, and therefore that its outcomes (the research published in our journals) provides insight into the power relations of our community and its norms. This brief commentary examines how peer review has come to occupy this paradoxical position in our profession, as an ostensibly neutral standard of how to assess what is good in research practice and as a recognised method of unfair discrimination. In this discussion I also explore why it is important to question peer review as a process, what the Journal of Business Ethics is doing to address the inequalities it can produce, and how its future might be somewhat different from its past and present.

### A Short History of Peer Review

Building on its long informal history of seeking out business ethics research wherever it is happening, Journal of Business Ethics (JBE) editors in chief have recently developed a formal commitment to diversify scholarship in its pages and the field (Freeman & Greenwood, 2016). All editors are now committed to paying particular attention to authorial representation related to geography, race, ethnicity, gender, and other modes of being, alongside ensuring research quality, the integrity of the peer-review process, and a sense of fairness in outcomes.^1^ This already goes well beyond the standard peer-reviewed journal commitment to being global in reach and relevance by encouraging paper submissions from any location, a generic purpose often emphasised in hortatory workshops at conferences providing normative ‘how to publish’ guidance. For JBE, this commitment means significantly more, in two very material ways: as an attempt to represent a much wider range of voices authorially, to encourage discussion of concerns and issues that are often marginalised or excluded from our field’s most prestigious journals; and ensuring the peer-review process is fit for purpose to enable that. If this can be achieved, then we should see benefits for the journal as an institution, and the field it represents. Attention to this aspect of our professional practice may be particularly important in making advances in research ethics, if we accept that the publication process is a part of that.

These efforts need not be read as a straightforward challenge to the standard Global North editorial process that JBE’s long success is built on. Peer review is a process which dominates many of our working lives, and it is therefore easy to assume it will continue forever as is. However, our current system of peer review is, perhaps surprisingly, a very recent development in academic publishing: most historians of science now agree that it only gradually became common practice in natural and social science journals in the 1960s and 1970s. In other words, peer review is not much older than this journal, yet it can appear unquestionably sovereign in its current binary form, defined by rejection or acceptance. This short anniversary commentary explores an alternative with specific reference to the possibility of a structurally fairer process both during and after review. Along the way, I explain what development editing means, its implications as an editorial commitment, and the possibility that it takes editorial practice beyond conventional peer review as a gatekeeping process (mostly designed to exclude). I also, along the way, question how helpful, or ethical, the provision of guidance such as ‘how to publish’ or tips are, usually communicated in workshops, for maximising the probability of navigating the peer-review process successfully.

In considering patterns of publication measured by geography, institution, or gender and ethnicity, we know that one of the key barriers to equality of access, and therefore to equality of voice, is an understanding of commitments on both sides of the peer-review process. As well as respecting authors, JBE promotes respect for the peer-review community, an aspect of which is recognising that considerable thought and work go into reviewing. Clearly only manuscripts that are worthy of peer attention should go into the review process; if they are not, the handling editor should return them to the submitting author, with some observations on why this is the judgement.

However, this ideal has to be reconciled with the recognition that peer review can be a relatively opaque process that not all members of the global research community are socialised into or familiar with. JBE has sought to reduce this opacity for a number of years, as in other areas of editorial practice such as integrity, through use of a dedicated Development Editor. The person in this role works alongside Section Editors, to identify submissions that are unlikely to be positively peer-reviewed in their current form, but satisfy one or more of these criteria:

1. The paper focuses on an issue, concern, or theme that is currently under-represented in the journal and/or the field of business ethics;
2. The paper author/author team has trained and works in a country or region that does not currently speak frequently enough voice through the journal’s pages; and
3. The evidence or argument presented are unconventional and suggest the journal and/or field lacks an important way of understanding simply because of its current conventions as to what constitutes knowledge or knowledge production—in other words, the manuscript is epistemologically or ontologically challenging.

Submissions to any section can be referred to the Development Editor for consideration of promise in any of these three areas. Referral may result in the editors desk-rejecting the submission with specific developmental feedback offered, perhaps encouraging submission to another journal after further development, or in the editors identifying ways to develop the submission so that it might be advanced to peer review. If the latter happens, the Development Editor will handle the paper through the peer-review process, with the support of the section editor, working with new and experienced reviewers who are encouraged to see the potential or promise in the manuscript. The rest of this commentary is an account of why this is important, how it can change the peer-review process, and what effects this shift in practice might produce.

### The Ethical Implications of Double-Blind Peer Review: Gatekeeping and the Power of Editors

The peer-review process has become considerably better known and understood since the beginning of 2020, when the COVID-19 pandemic brought all kinds of science research further into public debate than they had ever been. Journalists and news media now often specify where knowledge is in relation to the “gold standard” of publication in a journal after the process of submission, editorial reading, peer review, and revision for publication (with the implication that publication in certain journals means the knowledge is somehow better). Reporters and commentators now regularly refer to evidence and theory presented in “preprints”, signalling that the knowledge is not yet agreed or part of the formal record of what we know we know (with the implication that it cannot be fully trusted), and they encourage readers to engage with the idea that science is fungible and continually contested [with the implication that this is a good thing, even though it creates (un)helpful uncertainty]. These developments have helped to solidify the reputation of a specific kind of peer review as necessary for data or theory to be trusted. Ironically this has happened just as a number of colleagues in management and organisation studies have suggested that double-blind peer review is founded on unrecognised and unrewarded intellectual work [and is therefore a form of exploitation of those willing to contribute anonymously to the development of others’ work, as Lund Dean and Forray (2018) argue], is hopelessly ethically problematic because it lacks transparency [and therefore reproduces specific forms of unequal power relations, as Hugh Willmott (2021) suggests^2^], and likely to create ethically troubling inequalities (Lindebaum & Jordan, 2021).

These commentaries on our professional practice are fascinating in themselves. They also remind us that our current practice is exactly that—how we tend to do things at the moment. As the colleagues whose reflections I have cited here show us, there are significant problems with the peer-review system, despite the effort so many put into it. We need not return to the moment when editorial decisions were made without reference to anyone else, or to any other criteria other than those a single editor brought to the table. However, we do need to consider our disciplinary norms, as well as the political settings (Baldwin, 2018) and economic context (i.e. the historical and spatial relations) that encourage or obstruct this way of deciding what knowledge is (Gaudet, 2014). This is, I believe, exactly what the editors of this journal did in their short 2016 statement observing that the current route to publication *appeared* to privilege some forms of knowledge about business ethics, and obstruct “others” (using that term deliberately).

This is not a technical concern about demographic representation or geographical diversity; those are, at best, indications of what is wrong, rather than targets to hit that show we are somehow doing peer review right in an ethical sense. Rather, I would like to suggest that a “one-size-fits-all” approach to submissions can create epistemic oppression, the persistent presence of which can hinder an individual’s or a community’s contribution to knowledge production (Dotson, 2014). Equally importantly, if we accept epistemic oppression in any form—including a peer-review process that systematically favours or disadvantages knowledge produced according to certain temporary, situated, norms *that can take on a mantle of permanence or inevitability.* If we do that, we are committing an “unwarranted infringement on the epistemic agency of knowers” (Dotson, 2014, p. 115) in places and moments that are peripheral in a range of ways.

This observation, from Kristie Dotson’s feminist analysis of what peer review can mean in material terms for the knowledge we create, takes us into a complex ethical space. It implies that those of us in positions of power—editors in chief, associate or section editors, reviewers—all bear some responsibility for the oppression of a majority of colleagues. Political, economic, social, managerial, and educational structures are all clearly present in how we navigate peer-review systems (or fail to); what Dotson emphasises is that there are forms of exclusion that are *irreducibly epistemic*. In other words, knowledge may not become recognised as such as a result of the (questionable) epistemological norms that we follow. While resilient, as Dotson tells us, these norms are inevitably time-specific human constructions, like the current peer-review system, and are therefore open to question and change. We choose whether they, and their effects, persist or not, and what their effects are while they are with us.

Dotson proposes some questions that I believe the development editing role and its principles can benefit from at least asking. First, credibility: we begin from the assumption that all authors can be credible. In other words, from whom and where a paper submission originates has no bearing on how it is read in terms of its (potential) contribution to knowledge. This is, of course, something that is embedded in double-blind peer review—in theory. In practice, editors are not assessing submissions blind; if we accept what many colleagues experience, that “the rubber really hits the road” when the editorial role-holder assesses who has submitted the manuscript and where from, credibility is read into both of those things. Editors working collaboratively is one very practical way of encouraging the widening of credibility beyond individual, institutional, or geographical assumptions, especially if the editorial team is as diverse as JBE’s.

Second, Dotson argues that epistemic oppression is supported by epistemological resilience in the established order—the more stable the order, the more disturbance will be required to achieve change, and the more difficult it will be to generate the required level of disturbance. I have heard JBE criticised for publishing too much, as if knowledge were finite and conversations about it needed to carefully controlled. Constructing peer review’s primary purpose as exclusion seems to me highly problematic as an approach, especially if we consider the flaws and failings in all peer-review systems in terms of their ability to reach transcendental standards of “objectivity” and “quality”. Making space for a different kind of editorial work, oriented towards opening some of our gates rather than keeping them closed, may be a small contribution to undermining the epistemological resilience of the normative order.

However (and this is the final, somewhat pessimistic, point that Dotson makes), the key challenges in this aspect of knowledge production lie in getting those in positions of power to even recognise the possibility of epistemic oppression. This is, for me, the most valuable part of development work of this kind—it is a very visible, editorially supported, legitimate activity that implies all is not well epistemically, that we know it, and that we can do something about it.

### Conclusion: Back to the Future Through Development Editing

We have recently seen a series of practical proposals related to how to fix the peer-review system we labour with and under: rewards for acts of citizenship like reviewing, sanctions such as automatic rejection of manuscripts for failing to accept invites to review, more public recognition of review work, changes to the “blinding” process (including raising the curtain entirely post-acceptance). These are all thoughtful and clearly worthwhile, if the current system is as broken as some suggest.

This short commentary begins from a different position and provides a different approach. Peer review as we currently practise it is a highly specific response to a very particular set of conditions. It has developed rapidly, often in a technologically determined way (what did we do before photocopiers became relatively widely available, before the construction of online submission systems, and what will we do differently, better or worse, when the next technology arrives?), and through human endeavour. If we know anything about peer review, we know that it is temporary, changeable, and highly significant in terms of the knowledge we produce (or fail to put into the public domain). Editorial work has always been central to the life of a journal; development editing provides an additional way of seeing, diversifying voices and understanding, while respecting all community contributors to the construction of that knowledge.

It is difficult to predict what this future might look like. As recent contributions to our understanding of peer review show, critique is more straightforward than resolving the problems that current professional practice or epistemic norms create. This is further complicated by the recognition that those of us writing about this tend to occupy positions of relative privilege ourselves, having been successful enough within the current system to be granted space to critique it. As Dotson (2014) observes, fields such as ours can demonstrate remarkable epistemological resilience, excluding research that makes us feel uncomfortable. As so often, naming the problem is the beginning, rather than the conclusion; exercising social and political power in the service of a more representative, more inclusive, and more interesting range of research underpins the work between problem-recognition and a different sense of what counts as publishable research, in this journal and others.

## Awakening the Marketing Ethicist Within

**Michael R. Hyman**

### Introduction

To encourage broader participation in marketing ethics scholarship, this essay summarises a macro-level vision for creating ethics-centric manuscripts. After arguing that marketing ethics scholarship is available to anyone, basic research foci and storylines are suggested. Then a six-stage process and elliptical example are provided.

Most published advice about academic knowledge creation, whether general or discipline-specific, is generic and targeted at novice scholars. Hence it cannot help interested and well-trained scholars perform savvy inquiries into a subdiscipline (e.g. marketing pedagogy). To broaden participation in marketing ethics inquiry, this reflective knowledge creation essay summarises a macro-level vision for creating ethics-centric scholarship.

Marketing scholars can rely on formal arguments, scholar surveys, and literature surveys to identify, organise, and assess a subdiscipline’s ‘big questions’ (Hyman, 2004; Hyman et al., 1994; Skipper & Hyman, 1987). Formal arguments derive from first principles, scholar surveys aggregate scholars’ self-reports, and literature surveys analyse published work. Although helpful, these approaches are imperfect. For example, formal arguments may encounter an infinite regress problem (Skipper & Hyman, 1987), scholar surveys assume the wisdom of crowds rather than proneness to groupthink and other cognitive biases (e.g. availability bias, confirmation bias, wishful thinking), and literature surveys assume the past as prologue. To mitigate overconfidence in a single expert’s forecast, this essay focuses on a vision for identifying and assessing such questions, rather than propounding the most promising future research questions.

Although written from a veteran scholar’s perspective, research vision essays are not inherently egotistical. ‘Process knowledge’ acquired throughout an academic career is mainly experiential. Veteran scholars typically transfer such knowledge via apprenticeships with doctoral students and junior colleagues. This and future similar essays can augment that tradition.

### Knowledge Prerequisites for Marketing Ethics Scholarship

There are no knowledge prerequisites for pursuing meaningful marketing ethics scholarship. For example, my formal training is in the social sciences (i.e. economics, psychology, communications, and marketing) and not philosophy. Although this lack of formal philosophical training increased the challenge of assessing scholarship steeped in philosophical writings, ethics-related thinking need not be wedded to any philosophical perspective. Less-fettered thinking can yield more multidisciplinary and novel ethical observations and recommendations. Furthermore, an ‘outsider perspective’ befits marketing’s history of non-marketing-initiated thought leaders. Several influential marketing theoreticians migrated to marketing from other disciplines; for example, Shelby Hunt and Rick Bagozzi were undergraduate engineering students, and Jerry Zaltman earned his PhD in sociology.

No scholar would advocate philosophical ignorance; after all, intelligence and doctoral training do not negate the value of studying logical argumentation. Nevertheless, formal ethics training is nonessential for the many marketing ethics analyses, which require only substantive knowledge of the relevant marketing and marketing ethics literature. Often, a scholar’s moral compass can provide sufficient initial guidance, because it can point to intuitions and insights critical to identifying and exploring ethically problematic marketing ethics thinking and behaviour. However, when marketing ethics analyses demand philosophical expertise, teams of ethics-attentive marketing and philosophy scholars can collaborate. In a marketing ethics context, the goal of *unbounded collaboration*, defined as “the pursuit of scholarly research with one or more experts who have extensive scholarly training in one or more disparate disciplines” (Hyman, 1990a, p. 1), is to apply non-marketing expertise and perspectives to marketing problems. By offering non-marketing perspectives and improving interdisciplinary theory borrowing, such collaborations can broaden and buttress marketing ethics’ foundations via access to additional knowledge. (For an example, see Skipper & Hyman, 1993.)

Although many news consumers now prefer customisable and algorithm-refined online feeds to radio and television broadcasts, ethics-attentive marketing scholars tend to encounter the same stories about possible unethical or immoral behaviour. Journal and e-book aggregators (e.g. EBSCO), online search engines (e.g. Google), and self-archival websites (e.g. ResearchGate) ensure worldwide access to digitalised publications. Because ethics-centric analyses of marketing-related literature, news, and practice are not privileged (i.e. they do not require access to expensive or proprietary data and sources), the only prerequisites for marketing ethics scholarship are adequate graduate training in marketing and research methodology, a sound moral compass, and ethical attentiveness.

### Three Common Foci of Marketing Ethics Scholarship

Like most marketing scholarship, marketing ethics’ three non-mutually exclusive foci are theoretical foundation, practical application, and testbed for a comprehensive non-marketing theory. Although theory-centric inquiries may include a brief concluding discussion about implications for more ethical marketing practice, they generally focus on developing new theories or extending and improving existing theories. In contrast, practical application inquiries focus on societal deliverables (i.e. solving a societal problem such as reducing stereotypes or deceptive claims in advertisements).

Theoretical foundation-centric inquiries typical follow this general form:

Some marketing scholars believe ‘Theory X’. ‘Theory X’ is incorrect for at least one of the following four reasons: (1) It is contrary to ‘facts 1, 2, 3…’ (e.g. challenged by scientific anomalies: Kuhn, 1970); (2) Other marketing scholars believe ‘Theory Y’ instead, which ‘facts 1, 2, 3…’ support; (3) ‘Theory Y’ has greater explanatory power than ‘Theory X’, so ‘Theory Y’ supersedes ‘Theory X’ (Kuhn, 1970); and (4) Occam’s Razor suggests ‘Theory Y’ over ‘Theory X’. Thus, the solution is to reject ‘Theory X’ and adopt ‘Theory Y’.

Exemplars include inquiries that consider ways to augment codes of conduct for improved ethical decision-making, the ethicality of alternative economic orders, ethical corporate values and environments, and whether an ethical perspective pertains to resolving a marketing-related problem (e.g. Covid-19 messaging and care ethics). Given their often-esoteric nature, these inquiries’ influence generally is limited to academic audiences. However, their theoretical focus improves the likelihood of publication in prestigious scholarly outlets.

Practical application inquiries typically follow this general form:

Some marketing practitioners ‘do X’. ‘X’ is wrong because it creates preventable ‘harm Y’ (or fails to achieve ‘benefit Z’). The solution is ‘doing not-X’, mitigating ‘harm Y’, or adapting to ‘harm Y’.

Exemplars include inquiries that identify and address problematic advertising practices, like using deceptive appeals, encouraging celebrity worship, arousing fears excessively, ignoring racial stereotyping, or targeting children inappropriately. Such manuscripts may have public policy and litigation implications (e.g. Hyman & Tansey, 1990). Essentially, attempts to resolve practical issues typically reduce to the overall magnitude and distribution of benefits versus costs; for example, an advertisement is deceptive if the aggregate harm to all or the extreme harm to ‘one or more’ consumers or competitors is meaningful (Hyman, 1990b). Whereas failure to prevent ‘harm Y’ entails avoidable negative externalities, failure to achieve benefit ‘Y’ addresses opportunity costs.

As a practice-intensive discipline, marketing provides an excellent testbed for assessing comprehensive theories and measurement scales posited in other social sciences (e.g. sociology and the Theory of Social Character; psychology and the Theory of Reasoned Action). Unlike derisively characteried ‘theory of the month club’ inquiries, which reflect superficial and ultimately fruitless efforts to apply theory and applications developed in other disciplines (Jaccard & Jacoby, 2010), testbed inquiries can advance marketing ethics knowledge.

### Basic Storylines Related to these Common Foci

Although all marketing inquiries revolve around substantive and methodological storylines, the basic storylines associated with the aforementioned foci fall into three categories. (Note: Historical analyses can inform these storylines when the context is critical to understanding: Yan & Hyman, 2018.)

Type 1: ‘X’ is flawed but not broken, so tweaking rather than replacing ‘X’ is the answer.

Storylines of this ilk include creating new or improved ethical frameworks and practices, augmenting ethical codes and values, spotting ethically problematic behaviours and positing ways to discourage them, improving ethical training programmes, creating new or upgraded measurement scales, and improving marketing ethics pedagogy. Editorial teams are more likely to accept such storylines because they modify or extend rather than replace existing theory and practice (i.e. pose a lesser challenge to conventional wisdom). To motivate a tweak’s acceptance, marketing scholars should emphasise the marginal gains are sufficient to warrant it.

Type 2: ‘X’ is broken, and implementing ‘Y’ to fix or replace ‘X’ is the answer.

Type 2 storylines include encouraging ethical behaviour via suitable economics-related or regulatory (dis)incentives (e.g. decreasing unethical consumer behaviour by increasing its cost to consumers), new formal processes (e.g. minimising student cheating or stereotypes in advertisements), and informed decision-making rather than libertarian paternalism. When the broken ‘X’ is a theory, marketing scholars should ensure foundational consistency (i.e. the supporting nomological network relies on compatible underlying assumptions) (Skipper & Hyman, 1987). In contrast, when the broken ‘X’ is a practice, the cure must not be worse than the disease.

Type 3: ‘X’ is broken and unfixable, so adapting to this reality is the answer.

Type 3 storylines pertain to deploying mediational (e.g. addressing climate change by discouraging consumers’ global warming-related activities) versus adaptive strategies (e.g. ensuring adequate profits to digitisable content providers despite pervasive online piracy). To convince editorial teams, marketing scholars should argue that a ‘business as usual’ approach is unsustainable while resisting ad hominem attacks against proponents of conventional wisdom and practice. However, unsustainability arguments should avoid overly dire forecasts that trigger coping defences against extreme fear appeals.

### Six-Stage Process for Marketing Ethics Inquiries Related to These Foci

Rather than draw inspiration from the physics literature, Nobel laureate Richard Feynman preferred to find (or encounter) interesting problems and then derive solutions from first principles (Gleick, 1993). The following six-stage process takes a similar approach to marketing ethics scholarship. This approach is outlined and illustrated through an elliptical example.

Stage 1: Observe marketing behaviours that trigger ethical and moral intuitions.

Daily news reports bemoan the corrosive effect of pervasive misinformation on public discourse, behaviour, and well-being. Hence, theories and practices related to discouraging misinformation and defusing it once disseminated have marketing ethics importance.

Stage 2: Identify researchable questions based on those observations and intuitions.

Scholars cannot identify the right answers when they do not ask the right questions. Asking the right question in the right way often suggests “the answer”. Possible researchable questions related to Stage 1 observations are as follows:

- Can the perverse incentives that encourage disseminating misinformation be eliminated or mitigated (e.g. profits from hawking quack medicines)?
- Are efforts to reduce disseminating misinformation amenable to ‘broken window’ solutions (i.e. adapted from criminology, disseminating venial falsehoods may create a milieu that accepts society-damaging falsehoods)?
- Does susceptibility to misinformation differ by socio-economic profile variables and cultural differences (e.g. education level, individualistic versus collectivist orientation, political identity)? Does it differ across stakeholders?
- Does using moral suasion (rather than economic disincentives) to reduce misinformation dissemination represent an exercise in enforcing the unenforceable?
- What are the most effective ways misinformation disseminators inoculate recipients against counter-information?
- Do power imbalances between disseminators and receivers affect misinformation quantity and quality?

Stage 3: Ponder those observations, intuitions, and researchable questions until a formal argument emerges.

Like the Theory of Reasoned Action, but unlike Anne Elk’s theory of brontosauruses as told by the Monty Python comedy troop—they are thin at the ends and thick in the middle—the simplicity of this argument about information overload and disseminating misinformation should be an advantage.

Premise 1: In excessively data-rich decision environments (from a human information processing perspective), people cannot learn enough to protect themselves from many bad decisions and actions.
Premise 2: As a result, people generally adopt emotion-dominant (based on wishful thinking) rather than rational-dominant (based on logical thinking) decision heuristics.
Premise 3: Unethical actors exploit this heuristic-adoption tendency. Alternatively, ethical actors recognise this tendency and adopt an altruistic approach to decisions and actions.
Conclusion 1: Assuming caveat emptor often yields unethical decisions and actions in societies with excessively data-rich decision environments (from a human information processing perspective).
Conclusion 2: To encourage ethical thinking and action in excessively data-rich environments, altruism must replace caveat emptor as an organising principle.
Implication: Disseminators of society-damaging misinformation succeed because people adopt suboptimal decision heuristics in a globally overloaded information processing environment (i.e. they choose to believe the information they receive).
Stage 4: Consult a broad range of ethics- and behaviour-related literature (a) to inform and hone the argument, and (b) to verify if the current solutions are nil or insufficient.

Initially, the ethics- and behaviour-related literature suggests these non-exhaustive conceptual foci for an empirical study or argument-centred analysis on the ethicality of disseminating misinformation:

- A modern version of Glaucon’s challenge: “If I can get away with it and profit by it, why worry about whether it is ethical?” (Hyman et al., 1990a, b, p. 15)
- Caring about others (e.g. level 5 leadership; care ethics) (Shabbir et al., 2021)
- Caveat emptor versus altruism as the fundamental organising principle (relationship marketing and assuming repeated exchanges are more amenable to an altruistic orientation; a transactional model and assuming single exchanges are more amenable to caveat emptor.)

Stage 5: Conduct an empirical study or argument-centred analysis.

See Skipper and Hyman (1987) for a basic introduction to argument-centred analyses in marketing. Ideally, adequate graduate training and subsequent research experience in marketing and research methodology should be sufficient to conduct an empirical study.

Stage 6: Identify the best theoretical or applied solution(s).

If the research question(s) identified in Stage 2 is(are) non-trivial, identifying the best solution(s) before completing Stages 3 through 5 is impossible. In that grand scholarly tradition, expanding upon this elliptical example is left as an exercise for the reader.

### One Scholar’s Marketing Ethics Inquiries Yielded by this Process

Proving this approach is best for guiding marketing ethics inquiries is impossible. However, it has proven helpful in guiding one scholar's published marketing ethics research. The Table reflects a subjective grouping of the domains covered in a convenience sample of my ethics-centric publications: marketing communications (especially to vulnerable populations), negative externalities (unrelated to marketing communication), new or improved theoretical frameworks to enhance understanding and guide future research, a historical perspective, a testbed for comprehensive theories (e.g. Theory of Reasoned Action), new and improved measures, and improved pedagogical approaches (either about ethics or to behave more ethically). It suggests this approach can yield a diverse set of identified, pondered, and researched marketing ethics issues.

**Table:** Personal marketing ethics-centric retrospective

Research domain	Topics
Marketing communications (especially to vulnerable populations)	Ethically responsible advertising Deceptive and corrective advertising Advertising to vulnerable populations (e.g. host selling to children) Advertising sponsorship/advertorials (especially political) Racist imagery and stereotyping in advertisements Psychoactive fear appeal advertisements Ethicality and unintended consequences of wartime advertisements Unintended consequences of anti-child-abuse advertisements Promoting celebrity worship
Negative externalities (unrelated to marketing communication)	TV rating system Unintended consequences of consumption Online piracy Libertarian paternalism Unintended consequences of national pesticide bans
Conceptual	Ways to augment codes of conduct for improved ethical decision-making Ethical corporate values and environments Ethicality of the traditional versus the sharing economy Covid-19 and care ethics
Historical	Marketing and efforts to eliminate slavery in the U.K Puritans and the Protestant Work Ethic Framework for China-specific business ethics grounded in historical culture and values
Testbed	Moral judgements of salespeople (testbed for Forsyth EPQ)
Measurement	Multidimensional marketing ethics scale Virtue ethics scale Counteracting unethical response behaviour by survey participants
Teaching (either about ethics or to behave more ethically)	Approaches to teaching marketing ethics to undergraduates Ethical antecedents of cheating by students

## Business Ethics Informed by Feminist Economics

**Julie A. Nelson**

### Introduction

One might expect that the “Economics and Business Ethics” section of the JBE should be edited by an economist. Given that the economics discipline is still male-dominated, having it edited by a female and feminist economist may be less expected. Yet it was exactly my feminist work that motivated the editors—some years ago—to invite me to take on this task and motivated me to agree to it. Currently dominant conceptions about what a business “is,” I will argue, are simply saturated with a dangerously narrow and highly masculine-centric viewpoint. The field of business ethics has challenged this, and could challenge this more.

### Feminism and Economics

A great deal of feminist work in economics has—rightly—focused on the discipline’s exclusion of women as subjects of economic study. A substantial body of work has focused on discrimination against women in labour and financial markets. Care work—that is, work such as nursing and child care that has traditionally been primarily done by women—has received considerable attention, both when it is paid and when it is not. The critique of mainstream economic modeling and empirical techniques has been part of the feminist project, too. This has been particularly true when such techniques seem to especially distort the experience of women.

The central model of “the household,” for example, imagines it as a unitary entity. If one asks whose preferences are reflected in its choices, the answer is the household “head” (presumed to be male). Feminists noticed how this erases the existence of women (and children). We also examined models of the interior of the household that emphasise free choice and contracts between heterosexual marriage partners. Those tend to ignore very real differences in power determined by the larger environment of law and cultural norms, as well as the potential for conflict. Bargaining models of the household extended such models’ reach a bit—but only a bit.

And a number of us, at least, noted how economists’ neglect of issues of interdependence and power is rooted in its value system regarding appropriate methodology. The dominant belief in the discipline is that only mathematical models (generally, of optimal choice by rational, autonomous “agents”) satisfy the requirements for rigorous, scientific study. What to leave in and what to leave out is primarily decided on the bases of what would make the model mathematically tractable and the application of econometric empirical techniques possible. Anything that does not fit is ignored.

### What About “the Firm”?

The dominant model of “the firm,” unfortunately, has received less feminist analysis. Directly analogous to the model of “the household,” the primary model of “the firm” regards it as a unitary entity, whose “preference” is simply to achieve the highest profits possible. Models of the interior of “the firm” emphasise contractual relations in which labour power is (by mutual free choice) exchanged for a wage. I find the lacunae regarding interdependence, power, and the potential for conflict in “the firm” to be equally breath-taking, and, if anything, even *more* important for the survival of beings on this planet, than the analogous voids in “the household” models (Nelson, 2003).

Yet this idea of the firm as somehow essentially—metaphysically even—a site of profit (or shareholder value) maximisation remains extremely powerful. This image is, of course, of great service to those who want to personally amass and retain wealth and influence through (ethics-ignoring) business dealings. But I have also seen it take over the thinking of academics and other commentators who have no such interests to protect. I have seen its power within the pages of the Journal of Business Ethics, when an author (and apparently the reviewers and Section Editor as well) assumes that firms can act in an especially ethical way only when they can make a “business case” for doing so. An ethical action that would decrease profits is seen to be either impossible (because of overwhelming pressures from—supposedly—competitive markets) and/or to be welfare-reducing (because it interferes with the *summum bonum* of market efficiency). I have seen its power, too, in critiques of business coming from the political left and Marxist academics. They also mostly assume that the essential “nature” of the firm is maximisation of profits. It is only their conclusions that differ, in that they take this to mean that capitalist firms will always and everywhere exploit workers to the maximum.

Does everyone in a firm always seamlessly join together to do their best for shareholders? Do all the various shareholders have only *one* kind of interest? Do the choices of the executives (who are now, due to the advice of economists, often “incentivised” to focus on short-term stock movements) actually align with *anyone else’s*? Are people never interested in leaving behind a world their children and grandchildren can live in? Are markets always (or, some of them, *ever*) competitive? Is the *only* thing workers ever want from a job is the pay check? Is there just *one* kind of capitalism? It seems that economists’ models leave out a great deal.

The persistent dominance of a conception of “the firm” that has so many obvious shortfalls needs explaining. I believe that the answer lies in simultaneous and interpenetrating *gender essentialism* and *economic essentialism.*

### Essentialist Beliefs Vs. the Evidence

“Essentialism” is a long-established concept in feminist thought, and here refers to the idea that there are inherently two sexes with very specific characteristic preferences and behaviours, and very clear differences in roles. Essentialist thinking in Western countries posits that a (white and middle-class) male will manifest “masculine” characteristics, such as personal ambition, risk-taking, leadership, and strength in body or rational mind. He will “naturally” take on roles in “masculine” realms such as science, politics, and competitive commerce. The essence of being a (white and middle-class) female, in contrast, is believed to tend to manifest in “feminine” behaviours, such as putting another’s interest’s ahead of one’s own, avoiding risk-taking, and being cooperative and supportive. She is imagined to be suited for roles in the more “feminine” realms of the households or the study of the humanities, manifesting “natural” skills of nurturance, emotional intelligence, and caregiving. So-called “second wave” feminism of the 1960s and 1970s challenged gender stereotypes and concentrated on breaking the presumed ties between male/female sex and roles in society. Visible progress was made in the realms of economics and politics, though the effort to break the ties between “essentialist” gender and roles *within households* seemed to be less successful. More recent waves of feminist have noted how conceptions about gender also vary in important ways with race, class, and other factors, and some have challenged the notion of binary gender entirely.

But essentialism also applies more generally, because it is an energy-saving cognitive shortcut that our brains are very accustomed to using. As put by philosopher Sarah-Jane Leslie (and backed up by empirical cognitive science), “We essentialise a kind if we form the (tacit) belief that there is some hidden, non-obvious, and persistent property or underlying nature shared by members of that kind, which causally grounds their common properties and dispositions” (2017, p. 406). Such “underlying natures” are assumed to persist even in the face of obvious counter-evidence. As Leslie (2017, p. 406) explains:

For example, one might believe, implicitly or explicitly, that there is something about tigers that causes them to have stripes, to have four legs, to growl, to hunt their prey, and so on. These are not accidental features of tigers; they are grounded in the very nature of tigerhood. What is more, we believe that even a stripeless, three-legged tiger possesses this intrinsic, “essential” nature, even if she does not manifest its outward effects. The “essence” of tigers causally grounds these dispositions, though it does not guarantee their manifestation, since adventitious factors may intervene.

Applied to gender essentialism, this means that it does not really matter if one *observes* women taking risks or winning mathematics prizes, or men being generous or nurturing. These may have no effect on one’s bedrock beliefs (or cognitive habits) that set up opposing male and female “natures.” Dis-confirming observations are regarded as merely atypical variations around the general rule.

Likewise it does not matter if one *observes* business firms paying special attention to the well-being of their employees, initiating “green” measures, supporting legislation that would put a brake on greenhouse emissions, or pulling out of states that discriminate or countries that wage unprovoked war—even at the cost of current or future profits. It does not matter if one notes that a company’s shareholders are angry with CEOs for taking excessive compensation, or sees that a business leader is running a company into the ground. If one *believes* that “the essence of a firm is the maximisation of shareholder value,” then *evidence* is immaterial.

That is no way to run a social “science.”

While actual warm-blooded creatures roam the world, the notion that these divide “naturally” into men, women, and tigers is a cognitive artefact. While actual organisations buy and sell, produce and generate waste, and so on, the notion that these are profit-maximising-firms is a cognitive artefact. Cognitive artefacts such as these make our world seemingly easier to manage. As legal scholar Lynn Stout pointed out, shareholder value maximisation became popular in the business press because it provides “an easy-to-explain, sound-bite description” of what corporations are and do (2012, p. 19). But seeing our world only through the lenses provided by sound-bites and shortcuts can cause us to have massive blind spots and misconceptions.

### The Big Split

It is quite obvious—as feminist economists started pointing out in the early 1990s—that everything that characterises the mainstream discipline of economics aligns with dominant Western industrial society views of men and masculinity. These include its core definitions, subject matters, models, and methods. Rather than argue this point by point, let me illustrate with an example. Consider, the following statement of purpose adopted by the Econometric Society in 1930:

The Society shall operate as a completely disinterested, scientific organization . . . Its main object shall be to promote studies that aim at the unification of the theoretical-quantitative and the empirical-quantitative approach to economic problems and that are penetrated by constructive and rigorous thinking similar to that which has come to dominate in the natural sciences. (Roos, 1933)

The sort of thinking championed by the Econometric Society in 1930 has since come to dominate the entirety of the economics discipline. The masculine/science/tough slant (“We want to penetrate and dominate, too!”) has been characterised as “physics envy.” Economists’ theory of profit maximisation did *not* come from the observation of actual businesses. It was invented because it is a convenient and clever-looking way to represent “the firm” as a classical-mechanics-imitating calculus problem: max(profit) = revenue – cost.

Needless to say, in such a mechanical model of economic functioning, there is no room for actual, messy humans (of any gender) with our real feelings, vulnerabilities, and interdependencies, nor for social institutions in which real issues of communication, conflict, and the need for skilful management may arise. Where did these go? Consider this report on the first meeting of the American Sociological Society in 1905:

[We] are convinced that something is lacking in methods of interpreting human experience, and that the most effective means of supplying the lack must be sought without rather than within [the disciplines of history, economics, and political science]... The sociologists do not imagine that they are appointed to destroy the vocation of other investigators of society. They feel themselves called to represent factors in the problems of human association which have thus far received less than their share of attention… The society makes no appeal for credit. It simply proposes to encourage sociological inquiry and to await competent judgment of results. (American Sociological Society, 1907, pp. 579–580).

While sociologists at the time were mostly male, the language of this passage checks off “feminine” essentialist boxes all down the line: “We don’t want to offend anyone, or get any credit for our work.” Perhaps because venturing to study the social behaviour of people in businesses and markets might be perceived as encroaching on the turf of economics, sociologists seem to have largely refrained, at least until recently—the Economic Sociology section of the American Sociological Association was formed in 2001. As historian Mary O. Furner put it, sociology in the United States during much of the twentieth century ended up being made up of the “leftovers: marriage, the family, poverty, crime, education, religion, and sex” (Furner, 1975, p. 298). In other words, sociology got all the places where we are most obviously needy, where we most obviously do not fit the normative model of behaviour, where we are young and vulnerable, where we most obviously ask the big questions about our lives, and where we most intimately connect with each other.

### Business Ethics Straddles the Divide

The field of business ethics awkwardly straddles this old (imaginary, outdated, and extremely limiting) separation of the realm of commerce from the realm of social behaviour. For business ethics to not be an oxymoron, we have to acknowledge that business decisions could be motivated by real care about human beings and the future. We have to acknowledge that businesses are areas of human relations. We need to recognise that our workplaces, and not just departments of philosophy or religion, are locales where we work out (or not) the big questions about our lives and purpose. We have to notice that we do not leave our full humanity at the workplace door.

Such a project is, in many ways, the mirror image of feminist economists’ study of care work. Essentialist views of gender tend to sentimentalise activities such as child care and nursing, seeing them as arising freely from women’s “natural” compassion and tendency towards self-sacrifice. Feminist economists note that these activities involve both caring feelings and real hard *work*. As *work*, good caregiving demands time, energy, strength, education, skill, and financial compensation commensurate with qualifications and contributions.

Essentialist views of business and commerce reflect the opposite extreme, “tough-ifying” activities, such as corporate decision-making and market exchange. Business ethicists should note that these actually involve both work and an attitude of *care*. As areas of human social (not merely mechanical) interaction, good business and market relations demand attention to human vulnerabilities, long-term interests, and actual effects on well-being.

I have tried to come up with metaphors that might compete with the economistic image of economy-as-a-machine. With regard to business management, I have suggested revitalising the notion of “good husbandry,” that is, the bringing of attentive care to a productive venture (by leaders of any gender). Until economistic thinking took over, many would have thought it normal to expect that accepting a day’s pay would carry with it an obligation to actually put in a day’s work. In particular, it may have seemed normal that in return for a healthy salary CEOs would *do their jobs* and act as good stewards or nurturers of the enterprise, keeping it healthy and enhancing its long-term prospects. A leader who was simply personally greedy would likely have been looked at askance.

With regard to the economy, I have suggested the metaphor of a “beating heart,” since this organ is a circulatory pump, a living thing, and the metaphorical seat of both love and courage, all at the same time. Without care and innovation, it becomes diseased or dies (Nelson, 2018). Or we can observe that economistic assumptions infantilise people, imagining us as self-centred, insatiable, and irresponsible. To be sustainable, our world needs economies populated with more responsible grown-ups.

Essentialist thinking about gender and about business is lazy and damaging. My hope for the future of the field of business ethics is that it will get past both.

### The Economics and Business Ethics Section

I would like, going forward, for the “Economics and Business Ethics” section, which I edit, to get more submissions that I feel merit a review. I would like to see more insightful work that examines the long-neglected “problems of human association” *within business and commerce.* Such work would be especially appropriate for my section, compared with other sections of this journal, if it also references (probably contrasting) economics literatures. Or if it links careful analysis of business ethics issues with larger system-wide factors (e.g. regulation, competition, or economic development). Or if contains both an analysis of ethics and high-quality quantitative empirical work. These, I believe, could move the field forward.

Unfortunately, I currently get far too many submissions in which some “ethical” variable (such as some measure of “corruption” or “proportion of women on the board”) has been added to an econometric model, with little actual attention to ethical questions. These too often also tend to fail as quality empirical research, since as a discipline we have yet to adequately address the weaknesses and biases spotlighted by the “replicability crisis,” as increasingly recognised in the other social sciences and the sciences. In some cases, all empirical researchers “find” is their own pre-existing gender stereotypes (Nelson, 2014). The purely theoretical papers I receive in which an “ethical” variable is introduced into a mathematical model (of constrained optimisation or game theory) are usually similarly bereft of any useful ethical analysis. I generally reject such submissions without review.

When I get a paper that genuinely delves into ethics, I run some checks. I look to see if it seems to be written in a scholarly (rather than ideological rant) style; whether it may be of any interest to the business ethics community (rather than focusing on, say, some obscure bit of the history of economic thought); and whether the theoretical and/or empirical work seems (at first glance) potentially adequately explained and defended. Those that pass these screens I generally send to reviewers. I would appreciate receiving more such submissions.

## In Praise of Paraethics

**Gibson Burrell**

### Introduction

The argument herein concerns the possible development of paraethics which, by using the original Greek term ‘para’, I see in relation to ethics as being “beyond or distinct from, but analogous or parallel to”. By using the term ‘paraethics’, what I am advocating for undertaking serious and revelatory social science research is a move out from the confines of the notion of a universal code of ethics. In other words, I advocate a move from the ‘defensive’ ethics deeply enshrined within professional codes of practice to ‘progressive’ ethics. And at the heart of this version of progressive approaches would be paraethics. This entails, perhaps, being thought highly unprofessional! It might involve the embracing of covert activity with some subjects of interest, usually the powerful and relatively invisible (Meijl, 2005). It means moving in parallel to business ethics as we currently understand it, appraising it from the outside, and even, perhaps, moving off in a different direction when the need appears. It is an ethics closely allied to investigative journalism. It is likely to be dangerous and difficult work but extreme work of this kind is decidedly edgy (Granter et al., 2015). Yes, it will no doubt be scary, looking into the belly of the beast, but please consider what else is likely to raise your heart rates in the interest of ameliorative progress as you sit in your university office.

### Discouraging Research

In a seminar I attended at the University of Warwick given by a colleague from another university, he claimed that his former role as an investigative reporter had fundamentally shaped his research techniques. In his academic role, as well as his professional journalistic one, he announced that his primary objectives were to reveal the venality of the powerful and wealthy. His injunction to a room full of interested staff and students was to “get the bastards—by any means possible!”

This encouragement to move beyond accepted principles of ethical research caused a huge ruction in the seminar. There were those in the audience who thought that no scheme of research ethics should ever allow phone tapping or rifling through wastepaper bins or pretending to be something or someone that one was not. In robustly questioning the speaker, they showed their abject horror to such a worldview. Many of those in the audience were in the discipline of industrial relations, which at the time was characterised by a recognition of deep-seated socio-economic conflict, both in the UK and abroad. Yet these leaders in the field held back from “underhand” research techniques in revealing management intentions and strategies. They were highly respectful of legal arrangements, procedures and the rule book. To those who thought like me and found the speaker’s orientation very plausible, the staff in industrial relations were incredibly rule-bound and bureaucratically compromised. Nevertheless, these opponents of the “unmasking strategy by any means possible” won the day in the seminar room and, as it happens, across all disciplines, from the sciences to the social sciences and into the humanities. Techniques within investigative journalism and advocated by the speaker that day are supposedly banned from “science”.

Very recently, I chaired a PhD viva at a UK university. The candidate was studying “postfascist styles of organising” and had focussed his thesis upon analysing right-wing forms of social media rather than actually talking to people with such political and ideological dispositions about their views and their “take” on the aesthetics of the “Right”. When asked why he had not bothered to speak to the perpetrators of some of the hateful content, the panel were told that his University Ethics Committee refused several times to give him permission to do so. They thought he would be vulnerable to physical and psychological abuse, he might “go native”, and such a focus would only draw attention to these deeply problematic groups and their literature. Despite his pleas and those of his supervisors, we (through him) are not allowed proper access into this organisational world, despite its increasing global relevance. From my point of view, the University’s code of ethics has blocked our understanding of a problematic sphere with little “reason”, only a code that steadfastly refuses to accept the need for dangers of some kinds in important social science research. As the viva concluded, the candidate revealed that he now believed that “investigative journalism” was the only possible route for researching such activities.

Given these two anecdotes that circle around deeply problematic social worlds, it is the view adopted here that paraethics heavily relies upon investigative journalism (although by no means exclusively) and that this is a professional set of activities of which we need to know more. First, however, we need some ground-clearing work to be undertaken.

### The Problem of Motorised Morality Within “Defensive Ethics”

This is a commentary that is not against ethics, it is not anti-ethics, it is not about meta-ethics. What it is concerned with is what lies *outside and in parallel to* codes of ethics as understood both as professional codes within the sciences governing the activities of researchers, and as those documents that large corporations avow they follow as governing their social responsibilities and so on. In both cases these are constraints on action as well as calls to action. Within this commentary, therefore, an intimate and implicit link between politics and ethics will be assumed. My argument concerns a differentiation between “defensive” ethics and “progressive” ethics.

Both seem to be legitimate orientations. Defensive ethics appear to be hugely successful as a field orientation based on the protection of individual and corporate rights. These rights are defended by and enshrined within a set of legislative and administrative structures. This attempt at the defence of obligations is an essential activity in which important strides have been taken across a number of areas. These include, *but not exclusively,* a concern for “codes” of ethics which are important in articulating and enshrining such defensive routines which function to protect both the corporation and the individual. But in this safe realm of defensive ethics, it is recognised widely that professional and corporate codes of ethics are highly politicised. What they exclude, what they forget, what they claim to be unaware of, what they say is unimportant, are all very indicative of their function. They represent an absence in a presence and it is that set of absences that I wish to focus upon briefly. For such codes can all too quickly become servants of power. Codes of ethics, as we shall see, may provide alibis and eliminate ethical quandaries for those who draw them up. They produce a “motorised morality” that flies to the aid of the powerful when required.

Let me raise a question which we should not lose sight of and is best expressed, perhaps, as “for whom is knowledge now ethical?” My assertion is that the protection offered by ethics is distributed asymmetrically. In the British context and perhaps more widely (especially in the Anglophone world), the social science researcher today is very much curtailed in what they are capable of doing by the spread of the science and medicine-based codes of ethics (Komić et al., 2015). This curtailment seeks to protect the subject from terrible exploitation and physical and psychological trauma which were suffered in the past by many of those researched. As we all know, permission has to be granted by subjects before they are able to be questioned or implicated in research activity. And reasons need to be offered to explain for what purposes and by what means the overall research is being conducted. This sounds to most, if not all readers of JBE, as more than reasonable, of course. But, as we have already seen, if one is seeking information from subjects (and about subjects) who have clear and obvious reasons not to reveal their beliefs, motivations and activities to the researcher for financial or political reasons (among others), then social science is the poorer for it. The power structures behind and built into most codes of ethics—in academe as well as business—operating in the West in the twenty-first century, disproportionately favour economic and political elites.

Codes of ethics are very often calculative, measuring, controlling of numbers, legalistic and focussed upon the duties of the practitioner. The code establishes what has to be done in order for professional staff to do their duty of service. But duty is organisationally based and is often enough directed by the state. Almost exclusively, duty is seen from within the context of capitalism as an unquestioned context for all actions and almost never as a morally indefensible system of human organisation. It is one of capitalism’s great achievements in the twentieth century to successfully maintain, even today, that “there is no alternative”. Moreover, under this regime of accumulation, much analysis has been de-socialised, so that many commentators see ethics as an issue, not of the state and society, but of the I/me and the Other. Ethics becomes an issue of two parties in interaction and is therefore fundamentally transactional. Notwithstanding the complexity of some of these arguments based on non-systemic and non-structural analyses, these remain sociologically truncated and far too simple.

Codes of ethics are culturally and historically contingent. Moreover, they need to be complied with for them to have meaning. Arrow (1973, p. 315) maintains that, while a business code of ethics may be of use to a system as whole and may be of value to all firms that follow it, the more firms in an industry that stick to it, the more the value of cheating to individual firms there is. The robustness of codes of ethics is a cause of anxiety to many. In response to this concern, codes of ethics become instrumentalised, so that they are of pecuniary advantage to those that follow them. While this is usually the corporation, it may sometimes be professional groups. Thus, in the area of bio-engineering science, Häyry and Takala (1999, p. 171) reveal that in the early 1990s “Scientists between themselves decided to proceed cautiously and to avoid arousing further popular outrage under the cover of professional ethical codes”. They slowly motorised a morality in order to gain advantage.

With further regard to the utilising a “motorised morality” to come to the aid of the powerful, recently the University of Leicester published a *Dignity and Respect* policy that was produced as a statement on how staff should interact with others. Looking at the rhetoric of dignity and respect within the policy, one is meant to see all of the liberal values enshrined within it as truly positive. However, it was a policy based upon a particular, if popular, model of the organisation. It assumed a flat organisation with reasonable people running an unobtrusive hierarchy for a community which share all major values in common. It assumed a relatively harmonious collectivity in stasis, where misdemeanours are uncommon and senior management have all the interests of all staff at heart. Its unitarist assumptions, however, were deeply problematic. It was a de facto motorised morality that, once mobilised, allowed management to have no truck with any critique of their attitudes and behaviour towards staff. A great number of open complaints by staff against their managers were ruled threatening to the dignity of senior management. An Emeritus Professor has recently been stripped of this title by the University’s senior body and has had email access permanently denied for merely commentating upon University press releases. Close executive interest was placed in social media exchanges between threatened staff and their wide external readership, so much so that an external firm was employed to monitor this traffic, reporting back to senior management on what quantitative and qualitative impact it appeared to be having. It is difficult to see that this set of policies has maintained and maintains the dignity and respect for affected staff. It became a policy suite used to silence staff as best it could. In other words, it was a code of ethics that was indeed defensive. But it was defensive of senior management and *not* the rights of staff which it purported to be at the outset (and which was the line that the academic trade union understandably swallowed). But, in setting out on the road to be defensive of human rights, to protect rather than exploit one’s research subjects, the academic researcher can lose sight of being more progressive, even aggressive in their dealings with the world around them.

### “Aggressive” Ethics

In the academic area of business ethics, Jones et al., (2005, p. 9) claim “very often the disruptive, critical and hopeful aspect has been lost. Traditionally, ethics was a word that would stand against the practices of the day, and against the common sense that assumed that whatever is done must be. Ethics was … a critique of common sense.” It will be the contention of this commentary that this spirit of progressive ethics, based on critique, needs to be rejuvenated. And the way of rebuilding a critical edge to business ethics that is progressive in spirit and intent is through paraethics. That is, commitments to paraethics show a deep interest in ethics that stand in parallel to prevailing methods and assumptions about what we can and should do methodologically and politically. These methods are alternatives to the mainstream and may well be seen as “edgy” and dangerous—for they are.

If defensive ethics are problematic in these ways, then what of aggressive or *progressive ethics*? My view here is that paraethics is essential for maintaining progressive ethics that are edgy and dangerous and which deliberately use methods and tactics that defensive ethics would regard as beyond the pale. This commentary will end with some suggestions about the wider enframing of what would be possible within an approach based on paraethics, but here, in the text, one begins immediately to think of how ethical issues might be addressed outside of conventional business ethics by being taken into the narrow realm of investigative journalism.

By investigative journalism what is meant here is not the short, few-line stories typical of the popular press. Instead, what is emphasised is the use of longform, structural stories in which a narrative has been developed within a systematic chronology. This may not appear to take us away from an episodic approach to a topic with a clear beginning, a middle and an end. But these essentials to controlling academic case studies and their presentation as teaching aids are replaced by *deep monitoring with a critical edge*. The emphasis in investigative journalism is therefore upon depth of understanding, the constant surveillance of the object of study, and a starting point of critical intention. Some case studies in JBE approach this sort of stance in their presentations (e.g. Bontempi et al., 2021) but many in the general field of business ethics fall short on one or more of these dimensions, when compared to investigative journalism.

Most investigative journalists have undertaken detailed courses on how to gather their information from a whole variety of sources, many of which would be excluded by codes of ethics in the social sciences. They would also not appear in the best-selling methodology text books. Looking at such student texts, we are told often that we must treat information about a respondent as strictly confidential, that the researcher must not misrepresent the nature of the study, intrusive information should not be solicited, the self-esteem and self-respect of the subject should never be violated, and the respondent has an obligation to be truthful and honest in their responses. If investigative reporters were held to these sorts of ethical standards, we should be the poorer for it. On closer inspection, every single one of these injunctions that might well be parts of defensive codes of ethics would be broken by investigative journalism.

For example, revealing their intentions to subjects of interest is seen by such investigators as something one does very reluctantly indeed. These reporters are subject to harassment, threats and legal action by some ‘targets’ of their interest. And the use of ‘targets’ as a term is not unproblematic (although it is common in marketing) to ethicists. Once their stories have been written up and gone through quality-control procedures, there is then the issue of access to an audience. The right of access to the findings of investigative journalists is seen as a basic human right for which they will fight. Yet others present their work as “balancing the scales of justice” underlining ways in which the law and the legal system have been tilted so far in the direction of the powerful. However, today, freedom of information laws have been used to great effect and reporters will have been trained extensively in looking (by way of example) for illicit financial flows and other forms of “wrongdoing”. The websites of organisational forms involving investigative journalism are informative and reveal a web of closely interconnected not-for-profit forms of collective. One imagines this is a form of protection for progressive ethical entities.

Here below is reproduced just one strap line for one investigative group, but it symbolises the orientation of very many organisational forms in this field. We must note here, please, the emphasis on analytical depth, wrongdoing, and the extension of the time required.

ProPublica is an independent, non-profit, Pulitzer Prize-winning newsroom that produces investigative journalism in the public interest. It digs deep into important issues, shining a light on abuses of power and betrayals of public trust—and it sticks with those issues as long as it takes to hold power to account.

Some investigative journalists begin from the assumption that the provision of objective information can be an issue of life and death, literally. Revelations about medical scandals, for example, and the actions of Big Pharma immediately raise issues of the health consequences for many users. So does the poverty-inducing nature of many employment practices in the twenty-first century. One cannot envisage the welcoming of investigations of these practices within conventional codes of conduct where their defensive nature—of the perpetrators, not the workforce—would stonewall researchers really rather easily. Here, the role of gatekeepers is crucial. In a text from 1976, a period when the social scientist lived in a world in which the Milgram experiments were both welcomed and possible, ways of dealing with organisational agents who stood in the way of conducting detailed research appeared simpler.

“Entry can be gained in one of two basic ways; covertly, through disguise, manipulation, false pretence and other strategies of deception; or officially, through open and consensual negotiation with the gatekeeper. However, while the former tactics have been characteristic stratagems of investigative reporters, journalists, espionage agents, and muckrakers in general, the tradition for social scientists has been different” (Broadhead & Rist, 1976, p. 325).

Today, covert access is extremely difficult to justify and so the legitimate gatekeepers are in extremely powerful positions, certainly more powerful than 50 years ago when circumventing alternatives were professionally possible.

### Forms of Paraethics

Perhaps at this point in this commentary, we should use Broadhead and Rist’s quote to identify the question of what lies “beyond” or “parallel to” investigative journalism but still remains a form of “paraethics”? What other strands are possible to this “other side” of social science? Immediately in reading the pejorative term ‘muckraker’, one thinks, more positively, of the “whistleblower” as someone who works to a code of ethics unrealised by the corporation for which she or he belongs. Their parallel concerns carry dangers and complexities for their careers and even, though rarely, their lives. So too, as Broadhead and Rist suggest, does the work of the “spy” or someone who lives in deep undercover for many years. The British agent for the Soviet Union, Kim Philby, maintained in interviews that he lived a life in which parallel ethics had to be constantly maintained—at huge cost to him and his friendships (Macintyre, 2014). This is the point with which I began. Paraethics are dangerous. They bring on many changes. And perhaps, just perhaps, business ethics is in need of just such a transformation.

The anecdotal method of building my case here continues. Two years after the speaker at the University of Warwick seminar had scared half the audience by his ethics being “para” to theirs, I attended a private party well outside of academic life. I was surprised to find that the speaker was a guest there and, in fact, was the only brother of the host. The conundrum was that the host was self-affirmedly Jewish, whereas the speaker publicly maintained an Irish Catholic name and ancestry. On talking about my surprise to see him there at this particular party, and asking the highly personal question about his self-identification, the speaker explained that he had decided that, living in Manchester, an investigative reporter who was seen to be Irish would fare better than someone who was believed to be Jewish. I did not get a chance to ask him why he thought this. Note that he neither publicly nor privately renounced his own identity. He more quietly performed a different identity. Indeed, one might say, he extended his lack of transparency in his methods unto himself. His role range came, we might suspect, with a very high tariff. So my point is that the embracing of paraethics, understood broadly, rather than ethics, has serious consequences, some of which can be highly personal. It may mean leaving a community of scholars to whom one is attached if one feels that their methods are too conservative and defensive. Nobody then, should see this embrace of the range of paraethical approaches as easy and unfettered—for it will not be.

### The Geometry of Business Ethics

One final question that the reader may have is, why should we envision the geometry of business ethics so as to have paraethics as “outside” and merely parallel to ethics—and not as a vibrant “radical flank” fully emplaced inside the body of the work of many of us? Moreover, is this commentary not simply asking in essence for more courageous options to be taken by researchers whenever they can? If I may say so, these are defensive questions rather than aggressive ones.

Questions that are defensive of our current stance seek to retain a degree of comfort in holding on to the notion that we are doing the right thing most of the time and that, within the constraints of our area, we are doing our level best. On the other hand, aggressive questions, which both threaten the worthiness of what we do and lay siege to our comfortableness, are meant to be located in one or two places this commentary. Choose, if you will, one of three positions: first, is your possible argument that paraethics represents nought and signifies nothing; second, is the possible belief that paraethics is a small wing of ethics and has a legitimate place inside the fold? Third is my position. Paraethics represents an opportunity for an angle of attack upon the socially and economically powerful and cannot be circumscribed by the linear conventions of business ethics as primarily understood at this point in the twenty-first century. We need to *go back* to a time when it was legitimate for researchers to be sneaky, adept at undercover work and good at muckraking over the affairs of entrenched elites. That, dear reader, is my besmirched vision of one possible ethical *future*.

## Footnotes

1 This understanding of representational diversity includes non- or anti-normative paradigmatic or epistemological research.

2 This is a notably critical perspective on peer review, focused on how it generates anger in authors in particular. It is also notable in its neglect of other forms of affect embedded in the relational power structures the academic labour process is framed by, in what Ros Gill (2010, p. 231) calls the “small-scale, micro-negotiations of power in the academy” from situated positions of power and inequality. There is still plenty of space to analyse “our aching backs, tired eyes, difficulties in sleeping and … multiple experiences of stress, anxiety and overload”, should we wish to see them and acknowledge them as central to this process too.
